# Saccadic Alterations in Severe Developmental Dyslexia

**DOI:** 10.1155/2013/406861

**Published:** 2013-06-02

**Authors:** Stefano Pensiero, Agostino Accardo, Paola Michieletto, Paolo Brambilla

**Affiliations:** ^1^Department of Ophthalmology, Institute for Maternal and Child Health, IRCCS “Burlo Garofolo”, Via dell'Istria 65/1, 34137 Trieste, Italy; ^2^Department of Electronics (DEEI), University of Trieste, 34127 Trieste, Italy; ^3^Scientific Institute IRCCS “Eugenio Medea”, 33100 Udine, Italy; ^4^Department of Experimental and Clinical Medical Sciences, DISM, ICBN, University of Udine, 33100 Udine, Italy

## Abstract

It is not sure if persons with dyslexia have ocular motor deficits in addition to their deficits in rapid visual information processing. A 15-year-old boy afflicted by severe dyslexia was submitted to saccadic eye movement recording. Neurological and ophthalmic examinations were normal apart from the presence of an esophoria for near and slightly longer latencies of pattern visual evoked potentials. Subclinical saccadic alterations were present, which could be at the basis of the reading pathology: (1) low velocities (and larger durations) of the adducting saccades of the left eye with undershooting and long-lasting postsaccadic onward drift, typical of the internuclear ophthalmoplegia; (2) saccades interrupted in mid-flight and fixation instability, which are present in cases of brainstem premotor disturbances.

## 1. Introduction

Dyslexia refers to the inability to develop the capability to read, at an expected level, despite an otherwise normal intellect. The definitive cause is unknown, and the clinical spectrum is quite variable.

A wide variety of intracranial abnormalities have been described on both structural and functional magnetic resonance imaging, including increased or decreased frontal lobe activity and size differences in frontal gyri, cerebella, temporal lobes, and thalamic nuclei.

Various forms of ocular disturbances have been associated with reading disabilities. These disorders include exophoria, esophoria, amblyopia, and binocular dysfunctions [1].

Many pieces of evidence show that deficits in the perception of rapid visual information impact on reaction times (been longer) for eye movements [2, 3], hand movements, and vocal responses [4] in persons with dyslexia. Various hypotheses have been suggested to explain the presence of sensorimotor disorders in dyslexia [5].

The “magnocellular theory” postulates a deficit in the magnocellular neuroanatomy and neurophysiology [6, 7] that leads to deficits in fixation and eye movements [8–10], but some authors do not agree with this hypothesis [11].

The “fast temporal deficit hypothesis” focuses on the limited ability of persons with dyslexia to process rapid sequential auditory and visual stimuli. This could be related to the magnocellular theory [8] or to a basic sequential sensory processing deficit, unrelated to dysfunction of the magno cells [12, 13]. 

The “cerebellar-deficit hypothesis” suggests that many deficits associated with dyslexia, such as automatization [14], time estimation [15], and speeded performance [16], are caused by abnormalities within the lateral parts of the posterior lobe of the cerebellum [17].

Whether persons with dyslexia have ocular motor deficits in addition to their deficits in rapid visual information processing is still controversial. Some researchers have found differences in mean saccadic reaction time, stability of fixation, and number of regression movements between persons with and without dyslexia on nonorthographic tasks [2, 3, 18], whereas others did not find these differences [19–21]. The last studies seem more oriented to find longer latencies of saccades, at least in some stimulation conditions that require a very rapid sequence of visual information processing [5, 22, 23]. On the other hand, Raymond et al. [24] suggested that poor maintenance of gaze stability, rather than inadequate control over saccadic eye movements, contributes to reading difficulties in dyslexics.

That abnormal fixation and saccades may impair reading is already demonstrated. Most unilateral cortical lesions cause only minor eye movement problems, but the acquired ocular motor apraxia from bilateral frontal or parietal lesions can impair reading severely [25, 26]. Brainstem or subcortical lesions may cause more severe saccadic and fixation abnormalities: reading difficulty with progressive supranuclear palsy has been attributed to square wave jerks disrupting fixation and hypometric and slow saccades impairing scanning [27], but paresis of downward gaze also makes reading impossible.

Finally, Bucci et al. [28] detected that binocular coordination (disconjugacy) during and after the saccade in some dyslexics is worse than that of nondyslexic children. Moreover, dyslexics do not show the stereotyped pattern of disconjugacy (divergence during the saccade and convergence after the saccade). The conjugate postsaccadic drift is larger in dyslexics. Therefore some alterations of the saccadic dynamics, due to an involvement of the motorial generation of eye movements, are present in some dyslexic patients.

We present the case of a dyslexic patient where a clear subclinical saccadic alteration is present; this alteration, involving the medial longitudinal fasciculus (MLF), was not already pointed out in the scientific literature.

## 2. Materials and Methods

A male patient, 15 years old, afflicted by severe developmental dyslexia was submitted two times (at two weeks distance) to a saccadic eye movement recording.

The neurologic exam, auditory and somatosensory evoked potentials, electroencephalography, and encephalic nuclear magnetic resonance were all normal.

At the clinical ophthalmic examination, the natural visual acuity resulted 20/20 in both eyes, orthophoria for distance and esophoria for near, no other extraocular motility disturbance, and normal dioptric media and fundus. Only Visual Evoked Potentials (VEP) to pattern stimulation (vertical black and white bars at a spatial frequency of 6 cycles/deg) showed a slightly higher latency than normal values (P100 wave of 126 ms in both monocular stimulations).

Saccadic movements of both eyes during binocular viewing were acquired. The movements were recorded by means of an infrared bichannel probe using the *limbus tracking* technique. The signal was low pass filtered at 100 Hz and sampled at 500 Hz with a resolution better than 0.2 deg. The acquisition was made in mesopic light condition, with the subject sitting one meter in front of a stimulation bar (a semicircular horizontal array of 255 red LEDs). The patient's head was held still by a forehead band and a chin rest. The visual dot stimulus moved randomly in timing and position with a maximum amplitude of 30 deg in a visual range of ±20 deg.

After a semiautomatic identification of saccades based on a velocity threshold (fixed to 5 deg/s), the classical saccadic parameters amplitude (*A*), duration (*D*), latency (*L*), and peak velocity (*V*_*p*_) were automatically evaluated for each identified movement. In addition, *A*/*D* and *A*/*V*_*p*_ relationships (the “main sequence”) were summarized.

The *A*/*D* relation was calculated as a linear regression (*D* = *m*∗*A* + *q*), while for the *A*/*V*_*p*_ relation the fitting curve was derived from the function *V*_*p*_ = 1/(*α* + *β*/*A*), where 1/*α* represents the velocity saturation value for *A* → *∞* and 1/*β* corresponds to the slope of the best-fit curve for *A* → 0.

## 3. Results and Discussion

The saccadic mean latency (±SD) in the first exam was 164 ms ± 33 and in the second 162 ms ± 35. The normal values for ages 14–18 years in our laboratory are 198 ms ± 18. In our case, the saccadic latencies appear lower than those in normal children, and this does not agree with a deficit of the sensorial input, even if a slight increase of pattern-VEP latency is present. It must be considered that the visual stimulus for the saccadic test was a red LED, without a structural component, and the patient was very much stimulated to perform his best performance.

Therefore as other authors, we do not think that in all cases of dyslexia a difficult interpretation of the visual input must exist, but it is possible that alterations of the ocular motor pathway are present, also alone.

**Figure 1:**
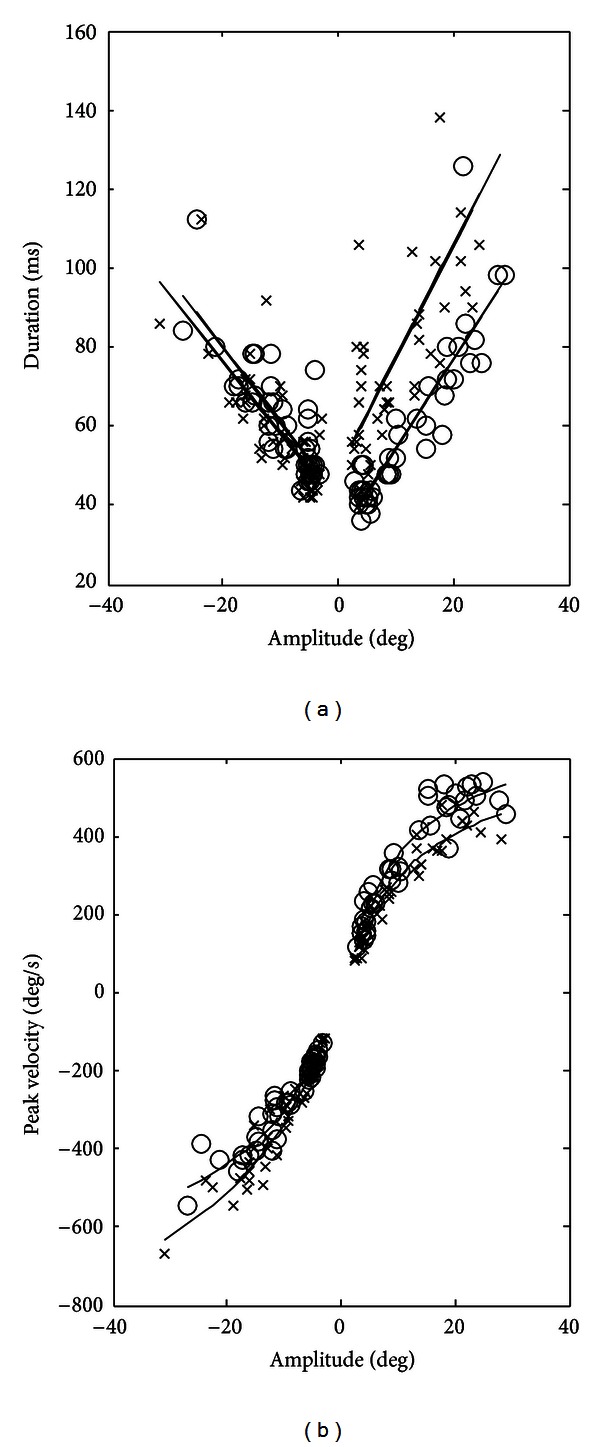
*A*/*D* and *A*/*V*_*p*_ relationships of the first exam. *⚪*: right eye; ×: left eye; in the *x*-axis the amplitude of saccades, negative numbers: movements to le left; positive numbers: movements to the right. A longer duration of the adducting saccades of the left eye is immediately easily visible.

The saccadic “main sequence” of the first exam is shown in Figure 1. A longer duration of saccades performed by the left eye to the right is evident (adducting saccades). This alteration was confirmed by the second saccadic recording. The regression parameters for the first and second saccadic tests are shown in Table 1.

At a brain stem level, a slowing of saccades can be due to an alteration inside the premotor system or the oculomotor nuclei, but a slowing of the adducting eye only is suggestive of a MLF involvement [29].

Lesions affecting the MLF cause internuclear ophthalmoplegia (INO). The cardinal sign of INO is paresis of adduction by the eye on the side of the MLF lesion during conjugate movements. The same eye may be able to adduct during convergence movements. In INO, impaired adduction can range from total paralysis to a paresis that is only apparent as slowing of adducting saccades. A commonly associated features is nystagmus on abduction of the eye contralateral to the lesion. Convergence eye movements may be preserved or impaired; acutely, an esophoria may be present, suggesting increased vergence tone. INO is also often accompanied by skew deviation, with hypertropia on the side of the lesion. Dissociated vertical nystagmus, downbeat with a torsional component in the contralateral eye, may be present. Asymmetry of vertical vestibuloocular responses (VOR) may be evident.

But patients with INO may have no visual symptoms, particularly when there is no limitation of adduction.

 Our patient presents only slight signs of unilateral INO: a slowing down of the adducting saccades of the left eye, without limitation of adduction, and an esophoria for near with good convergence.

**Figure 2:**
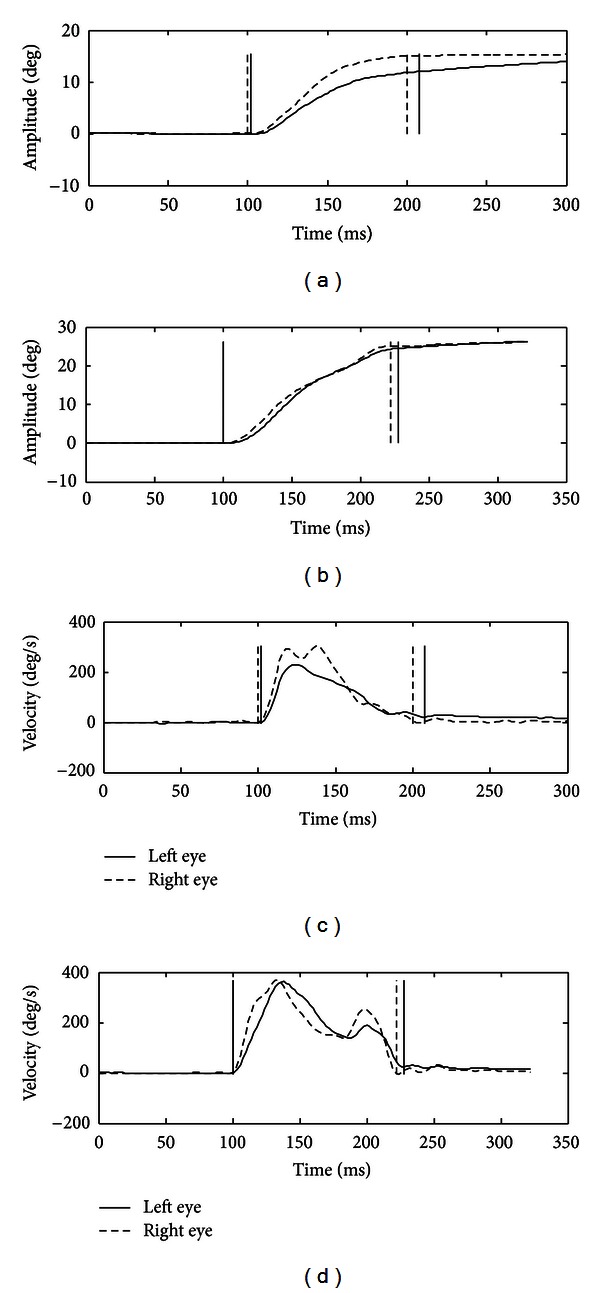
Saccades to the right. Upper graphs: eye positions, bottom graphs: velocity profiles. Left graphs: large undershoot of the left eye movement to the right with postsaccadic slow onward drift. Right graphs: saccade interrupted in mid-flight.

In Figure 2 (left traces), a saccade performed by the two eyes to the right is shown. A reduced peak velocity and a longer duration of the movement performed by the left eye (continuous line) are evident. This is due [29] to a pulse-step mismatch which is again present in INO: the step is larger than the pulse, and so the (left) eye drifts onward after the initial rapid movement (adduction lag).

Finally, the eye movement recording shows that intrasaccadic velocity slows down, present especially in movements to the right (Figure 2, right traces), as far as frequent instability of fixation at the end of saccades. Fixation instability, already pointed out in dyslexic patients, is present also in our case, and again it can be related to a motor pathology. Transient decelerations, evident on the velocity records especially of larger saccades, can be present occasionally in normal subjects. More often they are found in patients with disorders affecting the brainstem and cerebellum and are probably due to an incorrect function of omnipause neurons during a saccade [29].

Therefore, the presence of many saccades interrupted in mid-flight permits to postulate an altered function of omnipause neurons, which are part of the brainstem premotor system.

All these alterations, evidenced only by eye movement recording and analysis (been normal all the clinical and instrumental examination of the nervous system), show that in our case of dyslexic child a subclinical brainstem functional alteration of saccade generation is present, involving pulse generator and MLF.

## 4. Conclusions

Some oculomotor dysfunctions were unexpectedly found in a boy afflicted by severe developmental dyslexia. Similar subclinical motor alterations could be, alone or associated with other sensorial or motor impairments, at the basis of the reading pathology in other patients.

## Figures and Tables

**Table 1a:** (a)

*A*/*D* relation	First exam		Second exam	
	*m *	*q *	*m *	*q *
RE to the right	2.25	32	2.21	36
RE to the left	−1.89	42	−2.27	38
LE to the right	2.85	49	2.98	49
LE to the left	−1.83	40	−2.16	36

**Table 1b:** (b)

*A*/*V*_*p*_ relation	First exam		Second exam	
	1/*α*	1/*β*	1/*α*	1/*β*
RE to the right	730	68	822	55
RE to the left	−798	−49	−752	−54
LE to the right	662	53	685	44
LE to the left	−1100	−48	−894	−54

The normal values for ages 14–18 years in our laboratory for *A*/*D* relation are *m* = 1.87 ± 0.58 and *q* = 33 ± 6 and for *A*/*V*_*p*_ relation are 1/*α* = 1094 ± 308 and 1/*β* = 59 ± 18. In both exams only the 4 parameters of the LE to the right are out of the range of normality.
